# Innate Immune Pairing: Eosinophils as Hidden Architects of T Cell Immunity

**DOI:** 10.3390/cells14221826

**Published:** 2025-11-20

**Authors:** Kriti Gupta, Natalie A. Falta, Lisa A. Spencer

**Affiliations:** 1 Section of GI, Hepatology, and Nutrition, Department of Pediatrics, University of Colorado School of Medicine, Aurora, CO 80045, USA; kriti.gupta@cuanschutz.edu (K.G.); natalie.falta@ucdenver.edu (N.A.F.); 2 Gastrointestinal Eosinophilic Diseases Program, Department of Pediatrics, Digestive Health Institute, Children’s Hospital Colorado, University of Colorado School of Medicine, Aurora, CO 80045, USA

**Keywords:** eosinophils, T cells, intestine, lung, active immunity, barrier defense, homeostasis, oral tolerance, cancer, transplantation

## Abstract

Eosinophils, once primarily considered strictly end-stage effector cells in parasitic infections and allergic inflammation, are now emerging as vital immunoregulatory cells. This review focuses on eosinophil contributions to cell-mediated adaptive immunity by exploring the multifaceted interactions between eosinophils and T cells that underlie their unique contributions to immune modulation in allergic diseases. We begin by reviewing key features of eosinophil immunobiology within the context of their relevance to the development, differentiation, and function of CD4^+^ and CD8^+^ T cells in homeostasis and immunity. Building on this framework, we review recent literature revealing new roles for eosinophils in homeostatic immunosuppression, adaptive immune initiation, and immunomodulation within the context of an active immune response. We further explore the significance of eosinophil functionality impacting the structure and function of primary and secondary lymphoid organs, including thymic involution and regeneration, on cell-mediated immunity. This review presents an evolving paradigm that positions eosinophils as essential players in shaping multiple layers of the immune landscape in allergic diseases and beyond.

## 1. Introduction

Eosinophils are a subset of granule-containing white blood cells that were initially recognized for their association with helminth infections and allergic diseases and later appreciated more broadly for their roles in immune defense (including bacterial and viral infections and cancer) and tissue homeostasis. In healthy, non-atopic individuals, levels of circulating eosinophils are low (<5% of white blood cells), and eosinophils reside in diverse tissues such as the mammary glands, uterus, gastrointestinal (GI) tract, lung, and adipose tissue, wherein they primarily contribute to tissue homeostasis. In mice and humans, the highest density of steady-state eosinophils is found within the small intestine [1]. Despite their post-mitotic state, tissue-dwelling eosinophils rapidly adopt organ-specific adaptations that differentially equip them for tissue-specific functions [2,3,4]. In contrast to their steady-state homeostatic roles, within the context of eosinophil-associated diseases, eosinophils are recruited in excess into inflamed tissues, often with pathologic consequences. In both health and disease, tissue eosinophils may continue to be dynamically shaped by their microenvironment, as evidenced by distinct temporal and spatial niche-specific transcriptomes expressed by eosinophil subsets found within the same tissue [2,3,5].

Deposition of cytotoxic mediators and release of reactive oxygen radicals are well-known effector functions of eosinophils that cause tissue damage and exacerbate inflammation. However, the paradigm of eosinophil functionality has evolved to also recognize more nuanced contributions of eosinophils to tissue-resolving functions and immunomodulation. The extent to which eosinophil functions are instructed by their organ- and tissue niche-specific heterogeneity is an area of intense investigation. With the emergence and long-term usage of eosinophil-targeting biologics [6], there is renewed urgency in understanding the complex contributions of tissue eosinophils to health homeostasis, as well as disease.

This review focuses on eosinophils as immunomodulators, with an emphasis on their contributions to shaping cell-mediated immunity in health and disease, and within the framework of an evolving understanding of tissue eosinophil heterogeneity. We will review universal aspects of eosinophil immunobiology that uniquely equip these granulocytes for immunomodulatory functions, discuss organ-specific contributions of tissue eosinophils to maintaining steady-state immune homeostasis, and review recent studies that reveal novel roles for eosinophils influencing T cell functions in active immunity. Finally, we will explore the significance of eosinophil functionality impacting the structure and function of primary, secondary, and tertiary lymphoid tissues, including thymic regeneration, on cell-mediated immunity. Not surprisingly, the bulk of evidence revealing eosinophil immunomodulatory functions derives from studies in mice; therefore, our discussion in this review will be heavily weighted toward murine models, with human-relevant findings interjected where data are available.

## 2. Eosinophil Development and Tissue Heterogeneity

In the bone marrow, eosinophil development from CD34^+^ progenitor cells is orchestrated by a variety of key cytokines, principally interleukin 5 (IL-5), which expands eosinophil-committed progenitors and is the target of several eosinophil-targeting biologics. Although incompletely understood, IL-33 is likely another important regulator of in vivo eosinophilopoiesis, both through eliciting ILC2-derived IL-5 secretion and influencing eosinophil lineage commitment of ST2-expressing progenitors [7,8,9]. Eosinophilopoiesis is sequentially directed by key transcription factors such as Pu.1, c/EBPα, c/EBPε, IRF8, Gata-1, and GATA-2 [9]. C/EBPα promotes myeloid lineage commitment and inhibits Fog to prevent the suppression of eosinophil-specific genes, and c/EBPα-/- mice lack eosinophils and neutrophils [9,10,11]. GATA-1 instructs eosinophil-related gene transcription, including MBP [12], and IRF8 drives Gata1 transcription [13]. IRF8 deficiency reduces eosinophil precursors, and mutation of the Gata-1 promoter leads to loss of eosinophils in vivo [13,14]. Granule development is a requirement for eosinophil maturation, and interference with pathways related to effective granule formation and loading (e.g., deletion of the ER stress response-related transcription factor Xbp1, required for post-translational maturation of granule proteins [15], or dual deletion of the genes encoding granule proteins EPX and MBP [16]) also results in eosinophil deficiency. Eosinophil maturation involves upregulation of the G protein-coupled receptor CCR3, which facilitates their tissue recruitment, in large part in response to eotaxins (i.e., eotaxin-1 (CCL11), eotaxin-2 (CCL24), and eotaxin-3 (CCL26)), a family of (relatively) eosinophil-specific chemoattractants.

In steady-state, bone marrow-derived eosinophils home to tissue niches, including the lung, adipose tissue, and all regions of the gastrointestinal tract except the esophagus. Although transcriptome analyses suggest blood eosinophils exist as a relatively homogeneous population, analyses of tissue eosinophils reveal distinct organ-specific transcriptomes and maturation trajectories [3,17,18]. Eosinophil subset heterogeneity can be further observed within the same tissue, often in association with distinct spatial niches and/or within the context of local inflammation [4,19]. For example, eosinophils localized to the steady-state lung are morphologically and functionally distinct from those eosinophils recruited into inflamed airways. Perhaps more pronounced, surface receptor signatures distinguishing small intestine-adapted eosinophils, which house the greatest heterogeneity among eosinophil subsets in steady-state, include key immunoregulatory receptors, including PD-L1 and CD80. Therefore, the functional phenotype, including direct immunomodulatory potential, of tissue eosinophils is both organ and context-dependent.

## 3. Aspects of Eosinophil Immunobiology That Equip for Immunomodulation

It is worth noting that there are several aspects of basic eosinophil immunobiology that broadly equip tissue eosinophils for immunomodulatory functions. Below we will highlight their capacities to synthesize, store, and differentially secrete immunomodulatory cytokines, function as antigen-presenting cells (APCs), and engage in antigen-nonspecific cell–cell interactions that regulate T cell responses.

### 3.1. Secretion of Immunomodulatory Cytokines

Eosinophils secrete a diverse array of cytokines, T cell chemoattractants, and growth factors that contribute to shaping local tissue adaptive immunity. A cataloging of eosinophil-derived mediators has been reviewed extensively elsewhere [20]. Challenging their initial designation as strictly Th2-associated cells, eosinophil-derived cytokines include classic inducers and drivers of Th2 (e.g., IL-4, IL-13), Th1 (e.g., IL-12, IFNγ), and regulatory (e.g., IL-10, TGFβ) immunity. Likewise, eosinophil-derived chemokines include those with Th2 (e.g., CCL5, CCL17), Th1 (e.g., CXCL9, CXCL10), and Treg (e.g., CCL22)-recruiting potentials, revealing a broad capacity of eosinophils to initiate, polarize, and/or redirect a diverse range of immune responses.

**Figure 1:**
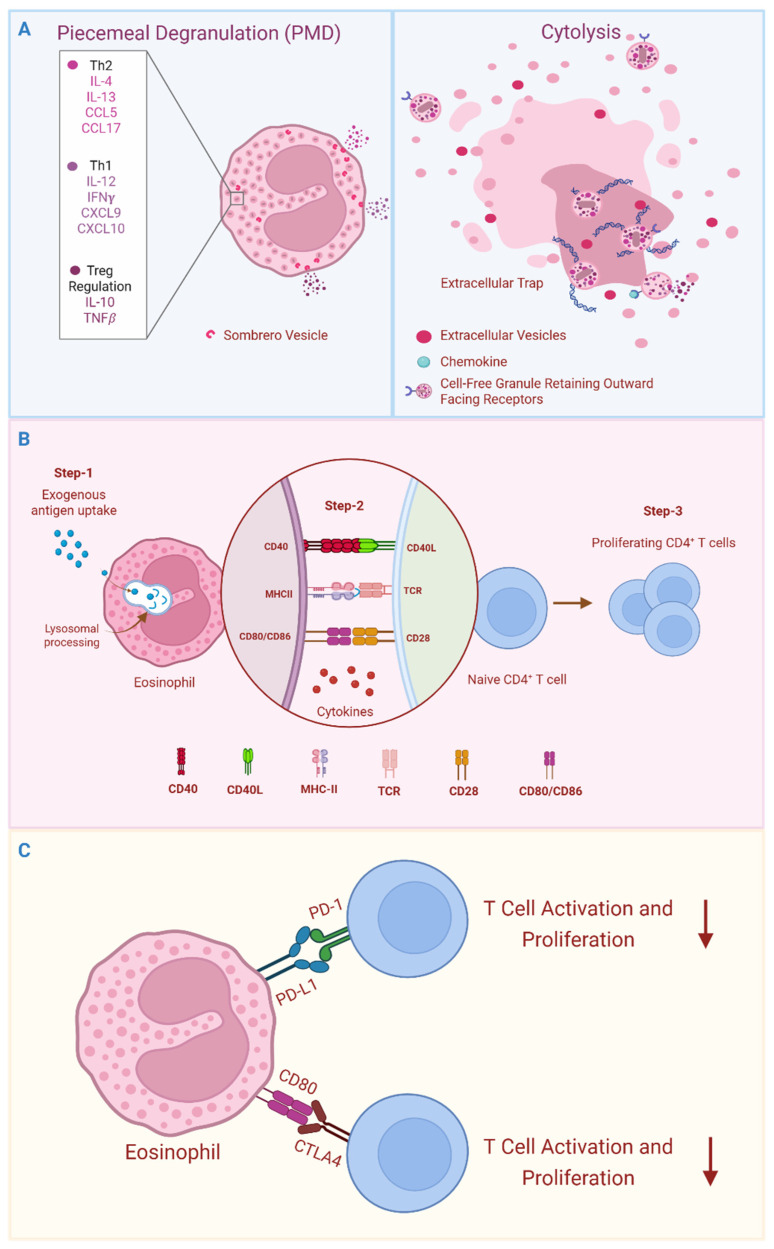
Attributes of eosinophil immunobiology are relevant to immunomodulation. (**A**) Eosinophil intracellular granules store a diversity of preformed cytokines, chemokines, and growth factors. Stimulus-dependent, differential secretion of granule-stored mediators is achieved through vesicle-mediated piecemeal degranulation (PMD) and deposition of intact granules and sombrero vesicles into the surrounding tissue through a process of cytolysis. Cytolysis can occur with or without extrusion of extracellular DNA traps. (**B**) Eosinophils can internalize exogenous antigens, which are processed within lysosomal compartments to generate antigenic peptides that are subsequently presented on MHC-II molecules. Stimuli such as GM-CSF elicit expression of co-stimulatory molecules such as CD80, CD86, and CD40 on eosinophils, enabling T cell co-stimulation, and secrete immunomodulatory cytokines to direct cell differentiation. Thus, eosinophil APC functions can facilitate antigen-specific clonal expansion and differentiation of naïve CD4^+^ T cells. (**C**) The local tissue microenvironment shapes eosinophil phenotypes, including expression of receptors with T cell modulatory functions (e.g., PD-L1), enabling antigen-independent regulation of T cell activities.

Like most other immune cells, eosinophils engage in de novo cytokine synthesis and secretion in response to diverse stimuli. In addition, eosinophils are distinguished from most other leukocytes by their storage of a diverse cache of cytokines, chemokines, and growth factors within intracellular secretory granules (Figure 1A). To date, over three dozen preformed cytokines and chemokines have been identified, warehoused alongside the cytotoxic granule proteins within intracellular granules of human blood eosinophils, poised for rapid secretion. In fact, human blood eosinophils emerge from the bone marrow with intracellular granules already stocked with a varied preformed mediator content that includes cytokines with dichotomous Th1, Th2, immune suppressive, and proinflammatory capacities [21]. Preformed and de novo synthesized cytokines are rapidly and differentially secreted from human [21,22] and mouse [23,24] eosinophils in response to distinct cytokine environments. In human eosinophils, physiological stimuli most often elicit a process of piecemeal vesicular degranulation, mobilizing a complex network of intragranular tubulovesicular structures [25] with embedded (non-signaling) cytokine ligand-binding chains that likely mediate differential cytokine sorting and trafficking (Figure 1A) [26]. Diversity of their preformed cytokine pool and capacity for rapid differential stimulus-induced mobilization reiterates the pleiotropic immunomodulatory potential already apparent in circulating eosinophils.

Release of eosinophil granule-stored cytokines is achieved via multiple mechanisms in response to environmental cues. In addition to the vesicular transport mechanisms of piecemeal degranulation described above, eosinophils undergoing cytolysis release tissue-deposited, cell-free granules and extracellular vesicles (EoSVs), either alone or in combination with DNA extrusion (i.e., eosinophil extracellular traps (EETs) that actively sequester microbes, Figure 1A) [27,28,29]. Eosinophil cytolysis has been detected in biopsies of patients with a variety of diseases, including eosinophilic esophagitis (EoE), dermatologic conditions, asthma, and intestinal bacterial infections [27,29,30,31,32]. Eosinophil cell-free granules liberated through cytolysis often maintain an intact delimiting membrane, presumably preventing nondiscriminant cytokine and cationic protein leakage within tissue. Functional outward-facing receptors and intragranular cell signaling molecules have been detected on eosinophil extracellular granules released through cytolysis, which were found to maintain stimulus-dependent secretion competence, suggesting preformed cytokines stored within tissue-deposited granules might continue to evoke immunomodulatory functions in the absence of intact eosinophils [33,34,35].

### 3.2. Eosinophils as Antigen Presenting Cells (APCs)

CD4^+^ T helper (Th) cells are activated upon T cell receptor engagement of their cognate antigenic peptide presented within the context of major histocompatibility complex II expressed on an APC, in parallel with receptor-mediated co-stimulation. A potential role for eosinophils as APCs had been hypothesized as early as the 1980s when the human MHC II molecule HLA-DR was detected on mature human blood eosinophils cultured ex vivo with granulocyte-macrophage colony-stimulating factor (GM-CSF) [36]. Not long after, HLA-DR expression was reported on sputum and bronchoalveolar lavage (BAL) eosinophils from patients with untreated asthma or eosinophilic pneumonia, respectively, and on BAL eosinophils from allergic rhinitis patients following experimental segmental lung allergen challenge [37,38,39]. In vitro, migration across an endothelial cell monolayer was also sufficient to upregulate HLA-DR on blood eosinophils [40]. In mice, eosinophils recovered from the peritoneal cavity of mice intraperitoneally (i.p.) infected with the filarial nematode Brugia malayi [41] or exposed to *Strongyloides stercoralis* [42] expressed high levels of MHC II. In parallel with MHC II, in vitro stimulated or in vivo activated human and mouse eosinophils were induced to express the co-stimulatory molecules CD80, CD86, and CD40 (reviewed in [43]). Of note, CD80 is expressed on intestine-adapted eosinophils in steady-state [2,44], and a subset of lamina propria eosinophils expresses low levels of MHC II [44].

Proof of concept that expression of the requisite machinery was, in fact, indicative of eosinophil APC functionality soon followed. Human eosinophils pulsed with staphylococcal superantigens [45] or exposed to tetanus toxoid [46] induced T cell proliferation. Importantly, in the latter study, T cell proliferation was abrogated if eosinophils were fixed prior to (but not after) tetanus toxoid exposure, suggesting antigen uptake and peptide processing by eosinophils. Human HLA-DR co-localized with the membrane tetraspanin CD9 within detergent-resistant lipid raft microdomains, a key attribute of optimal MHC II functioning in professional APCs [47]. Manipulatable mouse models demonstrated eosinophil trafficking to regional lymph nodes (LN) [48,49,50,51] and direct T cell engagement [52] and provided strong evidence of eosinophil APC functions in vivo and in vitro in both parasite infection and allergic airways experimental models [42,48,49,50,51,53]. Importantly, these findings included confirmation of antigen-specific activation of naïve CD4^+^ T cells by eosinophils [42,52], representing a function generally restricted to “professional” APCs, such as dendritic cells.

Antigen-exposed eosinophils were capable of internalization and processing of whole antigen through lysosomal compartments to generate antigenic peptides loaded into MHC II clefts [54,55]. An important experimental note: eosinophil exposure to lysosomotropic agents such as chloroquine or ammonium chloride-containing buffers traditionally utilized for red blood cell (RBC) lysis prevents eosinophil APC function, likely due to the requirement of lysosomal acidification for antigen processing [52,54,55]. Although eosinophils can acquire antigens from their surroundings through pinocytosis or phagocytosis, antigen engagement by Fc receptors enhances uptake by APCs, including eosinophils. In vitro, mouse eosinophils internalized higher amounts of OVA in the presence of OVA-specific IgG, an effect that was abrogated by inclusion of neutralizing antibodies to FcγRIII [56]. In vivo, small intestinal eosinophils acquired lumen-derived antigen in antigen-sensitized (but not naïve) wild-type (WT) mice, but failed to acquire luminal antigen in antigen-sensitized mice lacking immunoglobulin (μMT-/- mice) or Fcγ receptors (FcγR-/-), and passive transfer of antigen-specific IgG was sufficient to enable antigen acquisition by intestinal eosinophils in otherwise naïve WT mice [56]. Particularly relevant to atopic diseases, high-affinity FcεR1 has been reported on human (but not mouse) eosinophils, and in this issue of Cells, Wang et al. demonstrate enhanced IgE-mediated antigen uptake by eosinophils from humanized FcεRIa transgenic mice [57].

These data collectively provide proof of concept for eosinophil APC function and implicate humoral immunity in enhanced antigen acquisition. APC functions of eosinophils are illustrated in Figure 1B. It is worth noting, however, that although eosinophils *can* achieve the functional definition of “professional” APC in contrived human in vitro and mouse in vivo models, we are not aware of any definitive proof that non-redundant eosinophil APC functionality plays a meaningful role within the context of human disease.

### 3.3. Antigen Non-Specific Cellular Interactions

In addition to antigen presentation and co-stimulatory molecules, eosinophils can express additional receptors facilitating direct antigen-independent cell–cell interactions with immunomodulatory consequences (Figure 1C). One notable example is PD-L1, which functions as a checkpoint inhibitor, negatively regulating PD-1-expressing cells, including T cells. Eosinophil-expressed CD80 transmits a co-stimulatory signal through naïve T cell-expressed CD28, while CD80 engagement of CTLA4 on activated T cells transmits an inhibitory signal. Of note, PD-L1 and CD80 are constitutively co-expressed on a subset of intestine-adapted eosinophils in mice, readily observed in steady-state [2,44,58] (see Section 4.2) and increased within the context of microbial infection [30] (see Section 5.3), and PD-L1 is induced on lung eosinophils of asthmatics in response to IL-25 stimulation [59].

The following sections explore eosinophil-dependent modulation of T cell-mediated immunity within diverse tissue and inflammatory contexts, demonstrating how their diverse and complex contributions utilize these common aspects of their immunobiology, i.e., eosinophil secretion, APC function, and antigen-nonspecific cellular interactions.

## 4. Steady-State Tissue Immune Homeostasis

### 4.1. Steady-State Th2 Immunosuppression by Lung Resident Eosinophils

Lung resident eosinophils (rEos) patrol the pulmonary vasculature [60] and at least transiently enter the lung parenchyma of mice [61,62] and humans [62,63] in steady state. Lung rEos are morphologically, transcriptionally, and functionally distinct from eosinophils recruited to the airways following inhaled allergen challenges (i.e., inflammatory eosinophils, or iEos). Perhaps related to their largely intravascular association, lung rEos in both mice and humans maintain expression of CD62L, a surface selectin generally downregulated upon entry into tissues or cellular activation. Thus, CD62L has emerged as a potential biomarker to distinguish rEos (CD62L^+^) from iEos (CD62L^−^) within the lung [62]. iEos are further distinguished from rEos by upregulation of CD11c. rEos remain detectable alongside iEos within the context of inflammation, which may suggest they are neither converting into nor being replaced by iEos.

**Figure 2:**
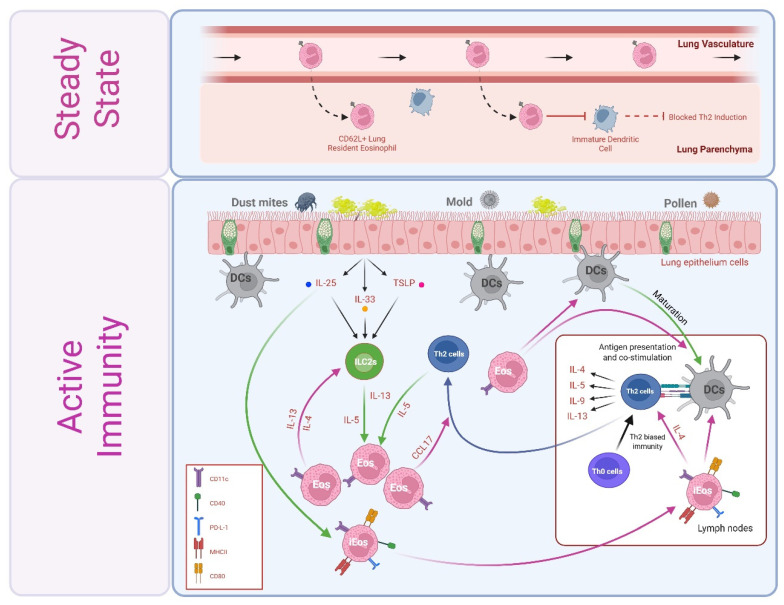
Eosinophil contributes to steady-state and type 2 immune responses in the lung. In the upper panel, CD62L^+^ eosinophils patrol lung tissue in a steady state and actively suppress type 2 immunity through inhibiting maturation and pro-Th2-inducing functions of DCs. Lower panel, in contrast, lung eosinophils recruited within the context of type 2 immunity exhibit an “inflammatory” phenotype (e.g., CD11c^+^) and may exacerbate a Th2-type response. Epithelial-derived alarmins, such as IL-33, IL-25, and TSLP, activate innate lymphoid cells type 2 (ILC2s), leading to the production of IL-5 and IL-13. These cytokines promote the expansion of eosinophil-committed progenitors in the bone marrow and facilitate the recruitment of eosinophils to inflamed tissues. IL-25 also enhances eosinophil expression of APC machinery. Eosinophils interact with dendritic cells (DCs) to enhance antigen presentation and co-stimulation of Th2 cells and promote DC migration into lymph nodes, thereby promoting Th2-biased immunity. Eosinophil-derived chemoattractants, such as CCL17, recruit Th2 effector cells into the lung tissue. In turn, Th2 cell-derived IL-4, IL-5, and IL-13 further drive eosinophil expansion and survival, exacerbating inflammation. Eosinophil-derived IL-4 and/or IL-13 may also regulate ILC2 populations, influencing their expansion and activity.

An immunomodulatory function of rEos was first suspected upon observing that eosinophil-deficient mice are more susceptible than their wild-type (WT) counterparts to developing allergic airway inflammation in response to low doses of house dust mite (HDM). Transient depletion of eosinophils during allergen sensitization failed to recapitulate the eosinophil-deficient phenotype, suggesting rEos, rather than allergen-elicited iEos, may mediate this basal immunosuppression. To this end, Mesnil et al. confirmed lung-derived rEos (but not lung-derived iEos nor intestinal tissue eosinophils) indirectly suppress Th2 inflammation by inhibiting the maturation and pro-Th2 inducing functions of dendritic cells in vitro (Figure 2) [62]. Th2 immunosuppressive functions of rEos are in stark contrast to the functions of inhaled allergen-induced iEos, which exacerbate Th2 immunity [64,65], as discussed in more detail in Section 5.1. rEos phenotype and function also differ from that of intestine-resident eosinophils, as described next in Section 4.2.

### 4.2. Steady-State Th1/Th17 Immunosuppression by Small Intestine-Resident Eosinophils

**Figure 3:**
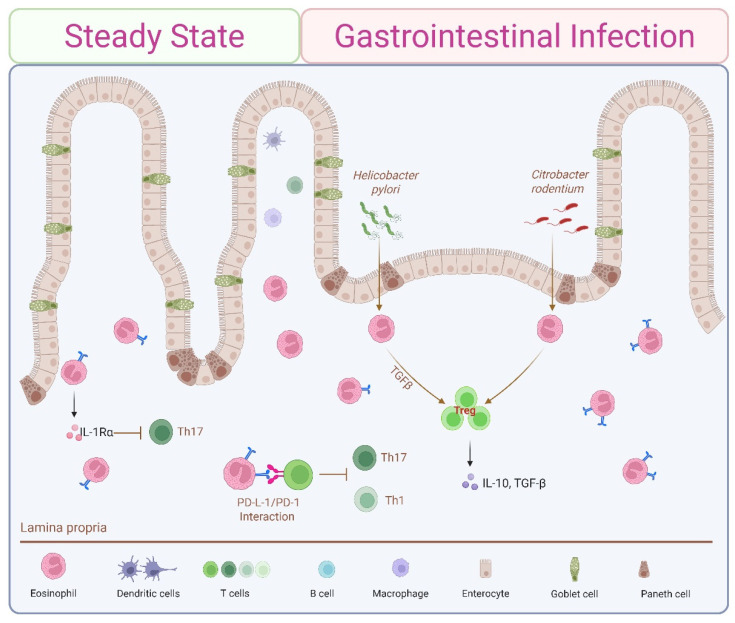
Eosinophil modulation of immune responses in gastrointestinal infections. Intestinal eosinophils restrict local immunopathology in response to microbial infection by suppressing Th1 and Th17 responses while promoting Treg cell expansion. Interaction with *H. pylori* or stimulation with IFNγ upregulates PD-L1 expression on eosinophils, enabling microbial-exposed eosinophils to suppress T cell proliferation through a PD-L1-dependent mechanism. Intestinal eosinophils also facilitate expansion of Treg cells, at least in part via TGFβ production.

In the steady-state gastrointestinal tracts of humans and mice, eosinophils are found within the lamina propria of all regions except the esophagus. Intestinal eosinophil subsets exhibit organ-specific adaptations, including expression of surface receptors directly associated with T cell modulation (i.e., CD80 and PD-L1) [2,3,58]. Intestine resident eosinophils regulate lamina propria CD4^+^Th cell immunity by actively dampening Th1 and Th17 responses. Eosinophil depletion (via genetic disruption, anti-IL-5, or anti-CCR3 inhibition) removes this regulatory effect, resulting in more aggressive Th1/Th17 immune reactions and increased inflammation in response to bacterial infection. Eosinophil-deficient mice exhibit a basal overexpression of Ifnγ and Il17 transcripts, increased expression of the Th1 and Th17 transcription factors Tbet and Rorγt, respectively, and an inverse correlation between numbers of small intestinal eosinophils and Th17 cells [66]. Reconstitution with wild-type (WT)-derived bone marrow or antibiotic treatment normalizes Th1/Th17 expression between WT and eosinophil-deficient mice, both confirming a role for eosinophils and implicating the microbiome. In contrast to the Th2 immunosuppressive functions of lung rEos (see Section 4.1), eosinophil deficiency did not alter steady-state intestinal Th2 responses, as indicated by unchanged GATA-3 expression and Th2 cytokine levels. In contrast to the elevated numbers of Th17 cells, eosinophil deficiency is associated with reduced basal numbers of their innate lymphoid counterpart, ILC3s, in steady state [67]. These steady-state immunomodulatory effects of intestinal tissue eosinophils are achieved in part by cytokine secretion (Figure 1A) and direct T cell interactions (Figure 1C). IL-1β is a key cytokine promoting Th17 differentiation by favoring alternative splicing of FOXP3 [68]. Eosinophil-dependent inhibition of Th17 is mediated at least in part by active secretion of the IL-1 receptor antagonist (IL-1Rα), a natural competitive antagonist of IL-1β, since eosinophils from IL-1Rα-deficient mice failed to suppress Th17 expansion (Figure 3) [66]. Attesting to the complexity of the system, GI eosinophils are also robust sources of Il1b, and eosinophil-derived IL-1β is implicated in maintaining sIgA and ILC3s [67]. Eosinophils also regulate Th1 activity directly via IFNγ-dependent expression of programmed death ligand 1 (PD-L1), which negatively regulates PD-1-expressing Th1 cells. Eosinophil-specific knockout of the interferon-gamma receptor (IFNγR) phenocopied eosinophil deficiency, confirming the central role of IFNγ [30]. As we will see in Section 5.3, PD-L1-mediated Th1 cell inhibition is further enhanced in microbial infections [2,69]. 

### 4.3. Eosinophil–CD8^+^ IEL Circuit Contributes to Intestinal Barrier Defense

**Figure 4:**
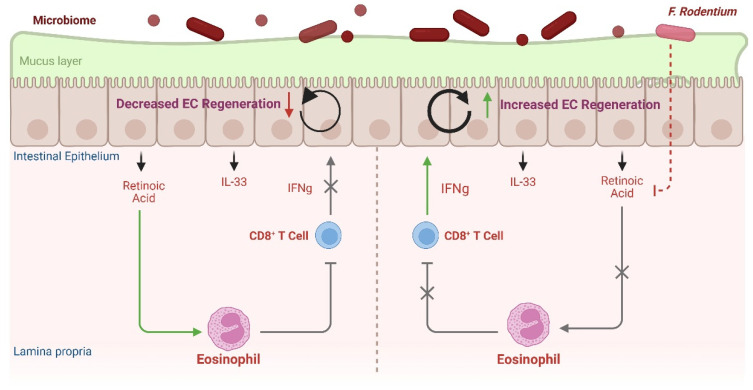
An eosinophil–CD8^+^ T cell circuit modulates duodenal epithelial cell (EC) regeneration. In mice, a subset of resident CD8^+^ IELs secrete IFN-γ, promoting EC regeneration. However, a retinoic acid (RA)-responsive eosinophil subset suppresses IEL-derived IFN-γ, inhibiting EC regeneration. The Gram-positive anaerobe *Faecalibaculum rodentium* reduces RA-producing enzyme expression in enterocytes, leading to decreased RA secretion and downregulation of RA-responsive eosinophils. This lifts IEL suppression, enhancing EC regeneration. These findings highlight a complex interplay between intestinal eosinophils and CD8^+^ IELs in epithelial barrier integrity.

In addition to influencing the CD4^+^ Th cell bias of the steady-state gut, intestinal eosinophils regulate CD8^+^ intraepithelial lymphocytes (IELs) that, in turn, contribute to the regeneration of the epithelial cell barrier. In contrast to the parenchyma-restricted distribution of lung rEos, steady-state intestinal eosinophils are distributed both within the lamina propria and in close proximity to the epithelial basement membrane [44]. Serving as a physical barrier, epithelial integrity is a critical front-line defense influencing immune regulation, guarding against inhaled or ingested allergens, invading pathogens, commensal microbes, and bacterial toxins. Epithelial cells (ECs) act as front-line immune sensors, rapidly releasing alarmins that serve as key early initiators of immune responses. Epithelial goblet cells secrete protective mucus layers of varied density, thickness, and functional properties and are further protected by antimicrobial peptides and secretory IgA. Villus blunting, fewer goblet cells and reduced mucus secretion, microbial dysregulation (particularly in mucus-dwelling microbes), impaired barrier integrity, increased GI motility, and decreased lipid absorption have all been reported in mice deficient in eosinophils [67,70,71,72]. Even temporary depletion of intestinal eosinophils induced by a high-fat diet was associated with increased intestinal permeability [73]. Many of these tissue homeostatic functions of GI eosinophils are driven at least in part by EC-derived mediators such as IL-33 and/or retinoic acid, elicited by microbiota-derived signals [72,74]. In one such microbiota-cellular immune circuit, Cao et al. demonstrated IFNγ from resident CD8^+^ intraepithelial lymphocytes (IELs) promoted intestinal EC regeneration (Figure 4). A retinoic acid (RA)-responsive eosinophil subset actively suppressed this IEL-derived IFNγ. The Gram-positive anaerobe *Faecalibaculum rodentium* reduced the expression of RA-producing enzymes in enterocytes, inhibiting RA secretion and downregulating survival of the RA-responsive eosinophils, effectively lifting IEL suppression and promoting EC regeneration [74]. It will be intriguing to delineate the “RA-responsive” eosinophil subset(s) implicated in Cao et al. within the context of a growing recognition of heterogeneous intestinal eosinophil subsets, including those intimately associated with the intestinal epithelium [44] and/or regulated by RA [75,76,77]. Taken together, these data implicate intestine-resident eosinophils in frontline homeostatic immunoregulation through the modulation of epithelial barrier integrity of mucosal surfaces, in part through regulating a subset of IFNγ-secreting CD8^+^ IELs.

Taken together, these data demonstrate immunoregulatory roles for tissue-resident lung and intestinal eosinophils in health and reveal overt differences between their steady-state regulatory contributions, with lung eosinophils selectively dampening Th2 immunity via modulation of DC activity, while intestinal eosinophils actively downregulate Th1/Th17 responses, at least in part through cytokine secretion and direct eosinophil-T cell interactions. The following section will address the changing spatial distributions and T cell modulatory functions of tissue eosinophils within the context of active allergic and infectious immunity and in specific examples of non-infectious immune responses (i.e., transplantation and cancer).

## 5. Eosinophil Contributions to Immunomodulation in Active Immunity

### 5.1. Allergic Airways

In a prototypical immune response to inhaled allergens (e.g., dust, mold, or pollen), epithelial-derived type 2-inducing alarmins (i.e., IL-33, IL-25, or TSLP) activate sentinel ILC2s. ILC2s produce copious amounts of IL-5 and IL-13 that expand bone marrow eosinophil-committed progenitors and indirectly promote eosinophil recruitment, respectively, and also promote Th2 immunity through direct and indirect actions on DCs and Th2 cells. Th2 cells recruited into inflamed airways secrete IL-4, IL-5, and IL-13, further promoting eosinophil expansion, recruitment, and survival. In contrast to lung rEos, iEos thus recruited in response to allergic airway inflammation in mice exacerbate Th2-biased immunity (Figure 2). Inflammation redistributes eosinophils to airway walls, wherein iEos-derived IL-13 elicits goblet cell hyperplasia and alters mucin composition to favor the inflammation-associated isoform muc5ac.

In addition to their well-recognized roles as downstream effector cells in type 2 immunity, several lines of evidence also implicate eosinophils as early immunomodulators. For example, although ILC2s are well recognized as essential drivers of eosinophil bone marrow expansion and tissue recruitment, eosinophil deletion reduced IL-13+ and IL-5+ lung ILC2s in a murine model of allergic airway inflammation induced by inhaled IL-33, through a mechanism requiring eosinophil-expressed IL-4 and/or IL-13. Complementary in vitro studies revealed that eosinophil-derived factors induce ILC2 expansion and chemotaxis [78], together suggesting a novel function for eosinophils in regulating ILC2s.

Eosinophils are also implicated in regulating Th2 cells, both directly through APC functions (Figure 1B) or secretion of T cell chemoattractants (Figure 1A), and indirectly through modulating DC function. As described in Section 3.2, eosinophils can take up and process antigen, migrate to draining mediastinal lymph nodes (mdLNs), and engage directly with naïve and activated T cells in LNs and local tissue, respectively [48,50,51]. Eosinophils from allergic asthmatics express higher levels of the receptor for the Th2 alarmin IL-25 [79,80], and IL-25 enhances their antigen uptake and HLA-DR and co-stimulatory molecule expression. IL-25-enhanced, Th2-inducing antigen-presenting cell functions of eosinophils were observed in vivo in an HDM model of allergic airway inflammation in mice and in vitro using autologous eosinophils and CD4^+^ T cells from allergic asthmatics [59]. In a mouse model of OVA-induced allergic airway inflammation, recruitment of myeloid dendritic cells (DCs) to mdLNs (and therefore subsequent CD4^+^ T cell activation, Th2 polarization, and expansion) was absent in eosinophil-deficient mice and restored upon adoptive transfer of eosinophils. Eosinophils in this model not only mediated DC LN accumulation but also specifically suppressed DC-mediated induction of Th17 cells. Eosinophil APC function was not required in this model, as transfer of MHC II^−/−^ eosinophils was sufficient to restore DC LN accumulation and subsequent CD4^+^ T cell expansion [64]. Of note, enteric eosinophils similarly regulated CD103^+^ DC migration to intestine-draining mesenteric LNs (MLNs) in a murine food allergy model and induced DC activation through secretion of EPX [81]. Activation and maturation of DCs by human blood eosinophils in vitro has also been reported, possibly elicited through EPX [81] and/or MBP [82] mediated effects. Within lung tissue, eosinophils further modulate the local T cell response; in a murine OVA model, eosinophils were required for the generation of the Th2 effector cell chemoattractants CCL17 (thymus- and activation-regulated chemokine) and CCL22 (macrophage-derived chemokine) [83]. Taken together, these studies reveal multifaceted contributions of eosinophils as immunomodulators through direct and indirect regulation of ILC2s, DCs, and Th2 cells throughout allergen sensitization, challenge, and effector phases of type 2 immunity (Figure 2).

### 5.2. Lung Microbial and Fungal Infections

**Figure 5:**
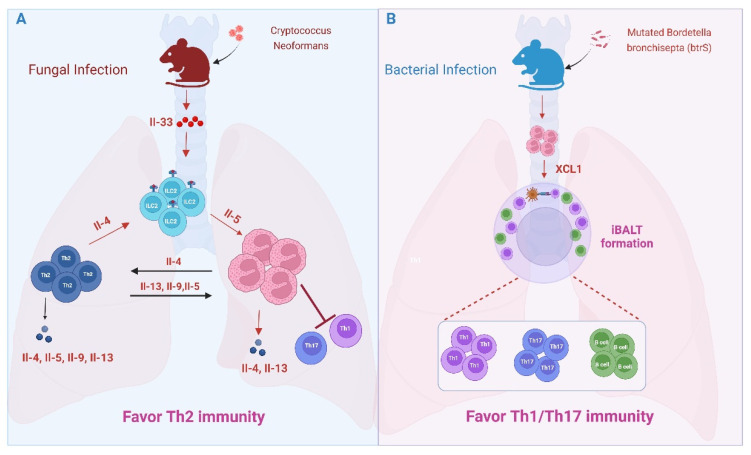
Eosinophil functions in lung infections. (**A**) IL-33 drives the accumulation of eosinophils and Th2 cells during *C. neoformans* infection. Both cell types produce IL-4, resulting in a Th2-dominated immune response that undermines protective Th1/Th17 immunity. (**B**) In response to *B. bronchiseptica* infection. Eosinophil infiltration is associated with the formation of inducible bronchus-associated lymphoid tissues (iBALTs), providing a local niche crucial for T and B cell activation, highlighting a novel role for eosinophils in the formation of tertiary lymphoid tissue.

As in allergic airway inflammation, eosinophils promote type 2 immunity in the lungs of mice infected with *Cryptococcus neoformans*. *C. neoformans* is a fungal pathogen causing cryptococcosis. Inhaled spores elicit IL-33-driven expansion of ILC2s and accumulation of Th2 cells and eosinophils. IL-4 reporter mice (4get mice) reveal eosinophils, along with Th2 cells, to be significant sources of local IL-4 that maintain a type 2-dominated immune response. Th2- and eosinophil-expressed IL-4 increased sharply at late-phase time points after infection, peaking at 42 days and remaining elevated up to 70 days after infection, overwhelming local production of IL-17 and IFNγ [84]. Unfortunately, a protective immune response against *C. neoformans* relies upon Th1/Th17 cells; therefore, eosinophil-derived IL-4 in this context undermines Th1/Th17 immunity (Figure 5A) [85]. A testament to the detrimental effects of eosinophils in response to *C. neoformans* and similar fungal pathogens, mice lacking IL-33, IL1RL1 (IL-33 receptor), ILC2s, or eosinophils reveal enhanced Th1/Th17 responses and improved antifungal immunity [84,86,87,88].

Despite their apparent Th17 suppressive functions observed in allergic airway inflammation and fungal models, immunomodulatory functions in the lung are not restricted to Th2 responses; rather, eosinophils can also promote Th17 immunity. This was recently elegantly demonstrated by Gestal and colleagues using the natural mouse pathogen *Bordetella bronchisepta* (Figure 5B) [89,90]. *Bordetella* spp. are Gram-negative bacteria that include human respiratory pathogens responsible for whooping cough. Like other *Bordetella* spp., *B. bronchisepta* utilizes conserved host immune evasion strategies that enable lifelong infection of murine hosts. One such strategy is mediated via the bacterial sigma factor btrS. Mutation of btrS rendered *B. bronchisepta* susceptible to rapid immune clearance in wild-type (WT) mice that was also associated with protective immunity against reinfection, providing an in vivo model system to dissect mechanisms of protective immunity. Immune clearance of the mutated strain was associated with eosinophil infiltration and robust Th1/Th17 and B cell responses. Intriguingly, eosinophil-deficient mice ΔbtrS mutants failed to elicit Th1, Th17, or B cell infiltration, suggesting a Th1/Th17 host-protective adaptive immune response is eosinophil-dependent. Eosinophils infected with the ΔbtrS mutants secreted IL-17A, which may contribute to their Th1/Th17-inducing functions [90]. Pulmonary infection in mice can lead to the formation of organized lymphoid aggregates directly beneath the epithelium. These so-called inducible bronchus-associated lymphoid tissues (iBALTs) provide a localized niche for T cell and B cell priming and activation. Effective clearance of the ΔbtrS mutants in WT mice was accompanied by the formation of lymphoid aggregates that resembled iBALTs, which were absent in the permissive eosinophil-deficient mice and dependent upon eosinophil-derived lymphotactin (XCL1), a dendritic cell and T cell chemoattractant [90]. These findings demonstrate Th1/Th17-promoting functions of pulmonary eosinophils and reveal a novel function of eosinophils in regulating tertiary lymphoid tissue architecture in microbial infection. This latter function will be discussed further in Section 6.

### 5.3. Gut Microbial Infections

As might be anticipated in light of the Th1/Th17 dampening effects of gastrointestinal eosinophils in steady state (see Section 4.2), intestinal eosinophils restrict local immunopathology in the face of bacterial infections, in part by suppressing Th1 immune responses and promoting FOXP3^+^ Treg cells (Figure 3). Infection with the gastric pathogen *Helicobacter pylori* elicits a robust increase in gastric eosinophils, T cells, and neutrophils, with eosinophils found within close proximity to T cells at the base of glands and throughout the mucosa. Numbers of bacteria-induced Th1/Th17 cells, and cytokines, including TNFα, IFNγ, and IL1β, are even greater in eosinophil-deficient mice, suggestive of an immunosuppressive role for eosinophils. In vitro, direct engagement with *H. pylori* upregulated eosinophil surface-expressed PD-L1 [30] and promoted TGFβ production [89]. Bacteria-primed eosinophils suppressed the proliferation of both WT T cells activated via non-specific anti-CD3/CD28 engagement and antigen-specific OTII T cells activated via OVA-loaded APCs through a PD-L1-dependent mechanism. PD-L1 expression on eosinophils was induced by stimulation with IFNγ, and eosinophil-specific deletion of the IFNγR abrogated their immunosuppressive effects [30]. Similar suppression of Th1/Th17 responses was seen within the context of self-limiting colonic infections of the attaching/effacing bacteria *Citrobacter rodentium* [30].

GI eosinophils also co-localized with and promoted expansion of FOXP3^+^ Treg cells, particularly those expressing RORγt, within the gastric mucosa of mice infected with *H. pylori* and colons of mice infected with H. hepaticus or *C. rodentium*. Eosinophil-derived TGFβ, readily upregulated by bacterial exposure, was required for local intestinal accumulation of RORγt^+^ Treg cells. Eosinophil deficiency had no impact on Treg priming and expansion within mesenteric lymph nodes (MLNs), suggesting eosinophils mediate a local effect on peripherally induced Treg cells in this model [91]. Of note, earlier work from Chen et al. demonstrates eosinophils from intestinal lamina propria (but not peripheral blood) are themselves uniquely capable of inducing differentiation of naïve T cells into Treg cells in vitro through expression of both TGFβ and all-trans retinoic acid (ATRA) [92].

### 5.4. Oral Tolerance

OVA allergen sensitization and oral challenge similarly induced eosinophil TGFβ expression and local intestinal Treg accumulation [91], a function that might also play a role in oral tolerance induction. Oral exposure of WT mice to a tolerizing dose of OVA prior to sensitization prevents a food allergic response. In WT mice, oral tolerance is accomplished in part by RORγt^+^ Treg cells [93], which is elicited in part through APC functions of RORγt^+^ ILC3s [94,95,96], and accompanied by a decrease in MLN Tfh cells and GC B cells, lowering IgE production. In contrast to WT mice, OVA tolerization failed to expand RORγt^+^ ILC3s or RORγt^+^ Treg cells in MLNs of ΔdblGATA1-/- eosinophil-deficient mice, which also exhibited higher numbers of Tfh cells, GC B cells, and IgE, and failed to generate immune tolerance [97]. Failure to expand MLN RORγt^+^ ILC3s in this model is reminiscent of their diminished presence in the steady-state GI tract [67] (see Section 4.2). Although further mechanistic studies are needed, these data suggest that eosinophils participate in the protection against food allergic responses through oral tolerance induction and that this may be mediated at least in part through their roles in promoting intestinal Treg cell development.

### 5.5. Systemic Microbial Infection

**Figure 6:**
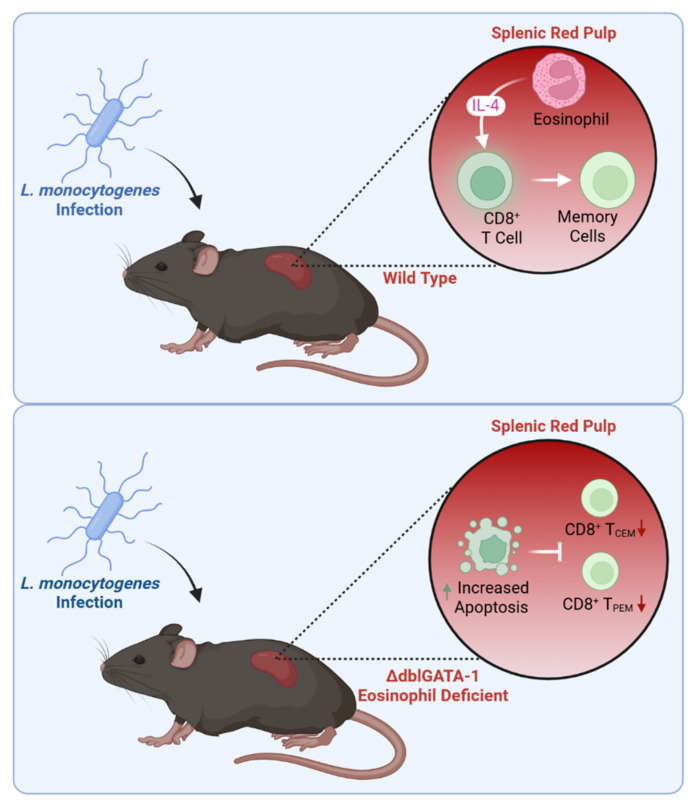
Eosinophils promote durable CD8^+^ T cell memory. Mice receiving a systemic dose of Listeria monocytogenes exhibited durable CD8^+^ T cell memory in WT mice (upper panel) that was abrogated in eosinophil-deficient ΔdblGATA1^−/−^ mice (lower panel). In WT mice, eosinophils located in the splenic red pulp provide IL-4, which protects antigen-specific CD8^+^ T cells from apoptosis. In eosinophil-deficient mice, increased CD8^+^ T cell apoptosis leads to impaired primary immune responses and reduced development of central and peripheral memory CD8^+^T cells, compromising responses to secondary infections.

Eosinophil immunomodulation is not restricted to the CD4^+^ Th cell compartment. The generation of memory CD8^+^ T cells is vital for eliminating pathogens and enhancing vaccine effectiveness. In a model of systemic dissemination of the bacterial pathogen Listeria monocytogenes, eosinophils were detected in close proximity to T cells within splenic red pulp regions within 2 days of infection, wherein they provided a local source of IL-4 necessary for the protection of antigen-specific CD8^+^ T cells from apoptosis. Eosinophil-deficient mice exhibited increased CD8^+^ T cell apoptosis, accompanied by a weakened acute immune response and impaired development of antigen-specific memory CD8^+^ T cells at later time points, with the most profound defects observed in central and peripheral effector memory cells (CD8^+^ T_CEM_ and T_PEM_, respectively), accompanied by impaired recall responses to secondary infection [98]. These data implicate eosinophil-derived IL-4 in memory T cell generation and systemic immune responses against infections (Figure 6).

### 5.6. Parasitic Helminths

Helminth is a term encompassing parasitic nematodes that include various taxonomic families and follow complex life cycles involving distinct developmental stages and tissue tropisms. Moreover, co-evolution with their human hosts has further complicated the task of generalizing the anti-helminth immune response, and eosinophils’ contributions to infection mitigation versus pathogenic outcomes are complex and context dependent. Despite their broad classifications, helminth infections, in general, elicit strong type 2 immunity characterized by eosinophilic inflammation, often initiated in much the same way as allergic inflammation, i.e., stress-induced type 2 alarmins activate and expand ILC2s, which in turn promote Th2 cell responses with accompanying Th2-biased cytokines (Il-4, IL-5, and IL-13) and eosinophilia. Along with other innate immune cells, eosinophils are an important primary source of IL-4 in response to larval helminth stages in some infection models (e.g., *Litomosoides sigmodontis* and *Nippostrongylus brasiliensis*) and contribute to the rapid establishment and continued positive feedback maintenance of type 2 immunity [99]. In contrast, eosinophils have also been implicated in suppressing Th2 responses. In the absence of eosinophils, mice infected with the enteric nematode *Heligmosomoides polygyrus* exhibited enhanced GATA3 and IL-4 expression by Peyer’s patch (PP) CD4^+^ Th cells, increased IL-4 secretion from T follicular helper (Tfh) cells, and exacerbated IgG1 class switching, relative to infected wild-type mice. How eosinophils suppress PP Th2 responses in this setting remains to be determined; however, the very low numbers of eosinophils within PPs might suggest an indirect immunomodulatory role, perhaps regulating local lamina propria CD103^+^CD11b^−^ DC subsets [100].

### 5.7. Non-Infectious Immune Responses

#### 5.7.1. Transplantation

Eosinophils are also implicated in immune regulation within the context of non-infectious tissue responses. Granulocytes, including eosinophils, are generally considered detrimental to organ and tissue graft acceptance. However, Krupnick and colleagues have demonstrated a *beneficial* role for eosinophils in facilitating acceptance of fully MHC-mismatched lung allografts in mice. Allograft acceptance was associated with enriched expression of the Th1-associated cytokines IFNγ and TNFα [101]. IFNγ elicits eosinophil-derived chemoattractants (e.g., CXCL9, CXCL10, CXCL11) and as described in relation to gut microbial infections in Section 5.3, upregulates eosinophil PD-L1 expression. Moreover, IFNγ and TNFα synergistically promote eosinophil expression of inducible nitric oxide synthase (iNOS) [102,103]. Eosinophil-mediated lung allograft acceptance resulted from both eosinophil-derived iNOS-dependent dissociation of the TCR/CD3 complex and direct contact-dependent T cell suppression via eosinophil-expressed PD-L1 and T cell-expressed PD-1 [101,103].

#### 5.7.2. Cancer

Eosinophils are known to infiltrate several solid tumors, particularly within mucosal tissues. In mice and humans, phenotypes of lung metastatic and colorectal tumor-associated eosinophils exhibited IFNγ and TNFα response signatures, primarily shaped by the tumor microenvironment (TME), and their presence was associated with enhanced recruitment of CD4^+^ and/or CD8^+^ T cells [104,105], presumably in part due to IFNγ-elicited secretion of T cell chemoattractants. Human colorectal or lung metastatic biopsies stratified by numbers of eosinophils showed enrichment of CD8^+^ T cell signatures in tumors with high eosinophils [104,105]. Likewise, in experimental mouse models of lung metastatic cancer induced by i.v. injection of breast cancer cells [104] or subcutaneous injection of colorectal cancer cells [103], genetic or pharmacologic loss of eosinophils was associated with diminished CD4^+^ (Th1) and/or CD8^+^ T cell recruitment into the TME; and in mice injected with melanoma cells in parallel with Treg ablation, activated eosinophils enhanced tumor rejection when co-transferred with tumor-specific CD8^+^ T cells [106]. By the same principle, emerging evidence suggests that eosinophils contribute to higher response rates of patients to immunotherapy. B-lineage non-Hodgkin lymphoma (B-NHL) patients treated with anti-CD19 CAR-T therapy showed stronger response rates in those with higher baseline peripheral eosinophil counts, and eosinophil depletion reduced tumor infiltration by CAR-T cells and impeded their anti-tumor efficacy in a pre-clinical mouse lymphoma model [107]. Immune checkpoint blockade (ICB) (i.e., through PD-1, PD-L1, or CTLA-4 targeting treatments) has become one of the most promising avenues of immunotherapy. In multiple cancers (e.g., triple negative breast cancer (TNBC) and non-small cell lung carcinoma (NSCLC), ICB was associated with the expansion of IL-5-secreting CD4^+^ T cells, contributing to eosinophil expansion and recruitment into the TME, which corresponded to increased expression of activated CD8^+^ T cell signatures in immunotherapy responders [108]. Using a mouse model of de novo mammary tumorigenesis that is refractory to ICB alone, this same group demonstrated a synergistic therapeutic effect of cisplatin and ICB that required eosinophils to achieve optimal CD8^+^ T cell activation within the TME [108]. Likewise, eosinophil molecular signals are increased in the TME following local radiation exposure (used to boost the effectiveness of ICB therapies) and correlate with endogenous CD8^+^ T cell infiltration and activation [109]. Of note, in contrast to the anti-tumorigenic contributions detailed above, mouse lung metastatic models of subcutaneous or i.v. injection of adenocarcinoma or melanoma cells revealed IL-5-driven eosinophilic tumor infiltration that was responsible for recruitment of Treg cells through secretion of the chemoattractant CCL22, contributing to a pro-tumorigenic TME [110]. Taken together, these studies collectively implicate eosinophils in tumor responses, at least in part through promoting T cell recruitment and/or functions.

Collectively, the preceding sections illustrate the multifaceted roles eosinophils play in regulating mature T cell subsets within both pulmonary and gastrointestinal tissues, spanning contexts of health and a wide range of disease states. The next section explores how thymic eosinophils contribute to shaping the naïve peripheral T cell pool, both by directly influencing T cell development and by supporting thymic tissue regeneration.

## 6. Thymic Eosinophils

### 6.1. Shaping the Peripheral T Cell Pool

Unlike other hematopoietic cells, T cell precursors complete their maturation process within the thymus. T cell maturation is a multi-stage, sequential process that begins with CD4^−^CD8^−^ (double negative, or DN) thymocytes entering the thymus and undergoing T cell receptor (TCR) gene rearrangement, followed by positive selection within the thymic cortex of those thymocytes expressing rearranged TCRs capable of recognizing self-MHC molecules. Positive selection elicits thymocytes expressing both CD4^+^CD8^+^ (double positive, or DP). These DP thymocytes migrate into the thymic medulla, where they interact with antigen-presenting dendritic cells and undergo a negative selection process that eliminates thymocytes with strong self-reactivity. DP thymocytes that survive negative selection downregulate CD4 or CD8 to emerge as either mature CD4^+^ or CD8^+^ (single positive, or SP) T cells, which are released into circulation and form the peripheral T cell pool.

In humans and mice, eosinophils naturally home to the neonatal thymus [111] and may contribute to the early life Th2 immune bias, in part through expression of indoleamine 2,3-dioxygenase (IDO), a tryptophan catabolizing enzyme that preferentially promotes Th1 cell apoptosis via production of kynurenines (KYNs, a tryptophan catabolite) [112]. Following the early life wave, the numbers of thymic eosinophils decrease with age, with the highest levels observed in children under five years old [112]. Providing yet another example of organ- and niche-specificity in eosinophil functional phenotypes, 30–50% of the human thymic eosinophil pool are CD34^+^ early eosinophil precursors [113], and phenotype heterogeneity among mature thymic eosinophils has been reported in both mice and humans (reviewed in [114]). DP thymocytes may influence these phenotypes, as eosinophil CD11c expression was markedly diminished in mice devoid of DP thymocytes [115]. In turn, thymic eosinophils may influence DP thymocyte negative selection and conversion to SP CD4^+^ or CD8^+^ cells. Eosinophils are detected within the medulla, cortex, and cortico-medullary regions, and in direct interaction with thymocytes [113]. In vitro, human thymic eosinophils promoted DP to CD4^+^ SP thymocyte transitions and actively diminished numbers of CD8^+^ SP thymocytes [116]. Studies in mice further support a role for eosinophils in thymocyte education and negative selection. Intraperitoneal injection of a self-peptide elicits an acute phase of thymocyte negative selection. Throsby et al. demonstrated that injection of an MHC I-restricted peptide rapidly elicited transient eosinophil recruitment into thymic cortico-medullary regions, wherein they were found in association with clusters of apoptotic thymocytes [117], prompting speculation of a role for eosinophils in clearing apoptotic debris and/or educating thymocyte deletion. Although direct APC function has been proposed for thymic eosinophils in mice due to their expression of MHC II and multiple co-stimulatory molecules [117,118], eosinophils do not appear to be involved with thymocyte negative selection in response to injection of MHC II-restricted peptides [117], and human thymic eosinophils reportedly lack MHC II proteins [116]. Although precise mechanisms remain to be determined, collectively these data implicate eosinophils in shaping the peripheral T cell compartment through influencing thymocyte negative selection.

### 6.2. Thymic Regeneration

In addition to their functions that directly shape the peripheral pool of thymic-derived T cells, eosinophils also play a key role in thymic maintenance and regeneration, enabling re-establishment of the peripheral T cell compartment after injury. Physiological responses triggered by various stimuli (e.g., infection, malnutrition, pregnancy, etc.) or therapeutic ablative interventions cause acute thymic damage that limits T cell output. Thus, thymic regenerative processes are crucial to restoring and maintaining immune homeostasis. Eosinophils are rapidly recruited in response to thymic injury induced by sublethal irradiation [119,120], and eosinophil depletion impaired both tissue regeneration and CD4^+^/CD8^+^ T cell output, thereby preventing post-irradiation reestablishment of the peripheral T cell pool [118]. Mechanisms whereby eosinophils promote thymic regeneration remain to be determined but may involve their contributions to apoptotic cell clearance [119] and/or positive feedback provision of IL-4 [120], a cytokine previously implicated in eosinophil contributions to liver [121] and muscle [122] regeneration. Of note, eosinophils were not essential for thymic recovery after myocardial infarction [123], suggesting their role in regeneration may depend on the type of thymic damage and/or the genetic background of the mice.

Together, these findings highlight the roles of eosinophils in shaping the peripheral pool of thymic-derived T cells, both through direct effects on T cell development and by facilitating thymic tissue regeneration.

## 7. Conclusions

Building from their unique immunobiology that includes storage and differential secretion of a diversity of immunomodulatory cytokines, antigen processing and presentation capabilities, and tissue- and context-specific expression of immune checkpoint receptors, eosinophils have emerged as important modulators of T cell immunity, both in health and across diverse disease states. Through direct and indirect modulation of ILC2s, DCs, and T cells themselves, eosinophils shape, dampen, and/or prolong T cell responses. In addition to long-established associations with Th2-dominant allergic and parasitic diseases, eosinophil-dependent regulation of cell-mediated immunity extends to Th1- and Th17-mediated responses and diseases ranging from microbial and fungal infections to cancer. Moreover, eosinophils are avid tissue repair cells, and substantial evidence exists to support novel tissue generative and regenerative roles for eosinophils affecting primary (e.g., thymus), secondary (e.g., Peyer’s patches), and tertiary (e.g., iBALT) lymphoid tissue structures. Further research is needed to fully elucidate context- and tissue-specific contributions of eosinophils to shaping the immune landscape in health and disease, particularly in humans. Moreover, increasing long-term use of eosinophil-depleting biologics that may effectively create “eosinophil-deficient humans” underscores the urgency of unraveling the complex immunomodulatory roles of tissue eosinophils while also presenting unique opportunities to study these functions directly in human tissues.

## Data Availability

Not applicable.

## Abbreviations

The following abbreviations are used in this manuscript:

GI	Gastrointestinal
ILC2	Innate lymphoid cell type 2
IL-5	Interleukin 5
MBP	Major basic protein
APCs	Antigen-presenting cells
EETs	Eosinophil extracellular traps
EoSVs	Extracellular vesicles
GM-CSF	Granulocyte-macrophage colony-stimulating factor
iEos	Inflammatory eosinophils
PMD	Piecemeal degranulation
IELs	Intraepithelial lymphocytes
RA	Retinoic acid
iBALTs	Inducible bronchus-associated lymphoid tissues
iNOS	Inducible nitric oxide synthase
TME	Tumor microenvironment
IDO	Indoleamine 2,3-dioxygenase
